# Assessment of agricultural region dynamics using object contour properties from Sentinel-2 images

**DOI:** 10.1371/journal.pone.0349631

**Published:** 2026-05-22

**Authors:** Serhii Nikulin, Kateryna Sergieieva, Oleksandr Kovrov, Anton Kozhevnykov, Olga Korobko

**Affiliations:** 1 Department of Information Technology and Computer Engineering, Faculty of Information Technology, Dnipro University of Technology, Dnipro, Ukraine; 2 Department of Ecology and Technologies of Environmental Protection, Mining Faculty, Dnipro University of Technology, Dnipro, Ukraine; University of Sindh, PAKISTAN

## Abstract

Economic activity in agricultural regions is influenced by various natural and anthropogenic factors. Their impact on spatial and temporal changes can be assessed by studying satellite images acquired at different times. The methodology for detecting changes is continually improving with the use of new image processing techniques. Changes resulting from destructive events can be used to assess their effectiveness. Agriculture in southern Ukraine has been catastrophically affected by military actions, which have led to the degradation of the agricultural landscape structure. One of the manifestations of this process is the destruction and fragmentation of linear contours of agricultural landscapes such as cropland boundaries, field roads, irrigation infrastructure, forest stands, etc. The study assesses the potential of using structural features of object contours, such as their total extent and contrast, in satellite image bands to monitor alterations in the condition of agricultural landscapes. A methodology is proposed to analyze the dynamics of cultivated land development or degradation by analyzing a developed Contour Density Indicator (CDI). The CDI was applied to assess changes in the configuration of an agricultural region in southern Ukraine from 2020 to 2024 using Sentinel-2 images. This period includes 2.5 years of military operations of varying intensity. Analysis of Sentinel-2 images demonstrated a systematic decline in CDI modal values from 2022 to 2023, with the strongest decrease observed within the 30-km-wide battle-line zone, where CDI mode dropped by up to 55% relative to 2020. In contrast, Ukrainian-controlled areas showed a partial recovery of CDI values in 2024, while the buffer zone continued to exhibit persistent loss of contour density, indicating sustained degradation of the agricultural landscape. The results enabled the identification of zones with signs of agricultural degradation resulting from depopulation caused by military activity and the subsequent decline in cultivation intensity. The methodology can be used for operational monitoring of agricultural regions and for making informed management decisions regarding land use in both wartime and peacetime.

## Introduction

Ukraine is one of the largest producers of agricultural products, accounting for 9–11% of global wheat exports and 13–16% of global corn exports. It is the fifth-largest global producer of corn and the ninth-largest global producer of wheat, respectively, from 2011 to 2022 [1,2]. The Russia-Ukraine war, which began on February 24, 2022, has led to production losses for each crop in Ukraine of more than 20% compared to before the beginning of 2022, according to some estimates [3,4]. As of 2024, due to the lack of economic activity in the fields of the battle line zone under constant threat of shelling, more than 18% of arable land in Kherson, Mykolaiv, Zaporizhzhia, and other Oblasts has been abandoned and is temporarily unsuitable for crop production [5,6]. Identifying and monitoring these areas is important for the economic assessment of crop losses from unplanted fields in the overall structure of the country's agricultural production [7].

Under war conflict leading to widespread depopulation and suspension of economic activity, there is a need for methods of large-scale monitoring of the state of agricultural landscapes [8–10]. Since ground-based observations in battle line areas are either impossible or unsafe, remote sensing is the only available source of spatiotemporal information on changes in abandoned lands [11–14].

Existing approaches to satellite monitoring of abandoned agricultural regions without ground-based verification data primarily rely on assessing the temporal dynamics of spectral vegetation indices, such as the Normalized Difference Vegetation Index (NDVI) and Vegetation Health Index (VHI) [15–18]. Change detection methods also play an essential role [19,20]. For example, the maximum seasonal NDVI of abandoned overgrown cropland is expected to be lower than that of cultivated fields [3]. To detect abandoned cropland based on secondary succession after abandonment, the Continuous Change Detection and Classification (CCDC) temporal segmentation algorithm can be applied to satellite image time series [21]. The Fallow-Land Algorithm based on Neighborhood and Temporal Anomalies (FANTA) method is used to differentiate between actively used and fallow agricultural land based on NDVI time series by comparing the current vegetation index value for a specific pixel with its historical values and indicators in neighboring pixels [11,22]. The dual-period change detection method identifies abandoned agricultural land by analyzing NDVI time series and comparing pixel-level vegetation curves for the pre-war and post-war periods [23]. Such approaches require the field contour mask, which is a time-consuming task. Furthermore, these approaches do not account for the structural changes that occur in anthropogenically organized agricultural landscapes in the absence of technological operations and crop rotation, particularly the loss of boundaries and contours. Quantitative assessment of the dynamics of contour density change can provide additional information about the degradation of agricultural landscapes and the presence or absence of economic activity.

The contours of land surface objects reveal not only the current spatial organization but also complex environmental processes such as succession, degradation, or recultivation of agricultural systems [24–27]. Changes in contours under reduced anthropogenic impact, such as depopulation due to war, may indicate a transformation of the landscape from a managed to a spontaneously evolving state. The physical mechanism of such changes in agricultural landscapes is described by the edge effect at the boundaries between different ecosystems [28,29]. Ecotones form at field boundaries, transitional zones between ecosystems characterized by specific environmental features, including microclimate, soil properties, and biodiversity [30]. Enhanced seed dispersal, temperature fluctuations, and fluxes of energy and organisms create unique conditions in such edge zones [31]. Under stable land-use conditions, ecotones inhibit the processes of biodiversity interpenetration between adjacent areas. However, as anthropogenic impacts decrease, these barriers break down, contributing to the disappearance of boundaries and changes in landscape mosaics [32,33].

The spatial proximity effect offers additional insight into the spatial dynamics of landscapes, whereby the proximity of areas with different land use types leads to their mutual influence on ecosystem processes [34,35]. The destruction of boundaries contributes to an increase in spatial interpenetration, a reduction in differences between land parcels, and, consequently, a loss of landscape structural stability and the degradation of agricultural land.

The study of contour density using Sentinel-2 data can help identify changes resulting from degradation, recultivation, or land-use change. Under limited access to in situ data, this approach is relevant for analyzing war-affected areas [36].

Existing structural change detection approaches in remote sensing primarily rely on spectral indices, pixel-wise change detection, or land-use classification schemes [37]. Despite their effectiveness in detecting categorical transitions, these methods are often limited by their high sensitivity to seasonal variability and the requirement for predefined classes or extensive training datasets, particularly in recent machine learning and deep learning approaches [38,39]. A critical research gap remains in detecting subtle, gradual structural degradation, such as the blurring of field boundaries or the loss of rural infrastructure, even when land-cover categories remain formally unchanged. This is particularly evident in regions experiencing reduced anthropogenic activity or conflict-driven abandonment, where spectral signatures may remain stable despite significant structural simplification.

To address this gap, a contour-based approach was applied, in which the density and contrast of object boundaries were analyzed directly from Sentinel-2 imagery without the use of training data or explicit object delineation. Based on these calculations, quantitative structural changes associated with landscape degradation and recovery processes were identified.

The objectives of this research are:

(a) to quantify the relationship between object contour density and the structural condition of agricultural landscapes under varying anthropogenic pressure;
(b) to develop the Contour Density Indicator (CDI) as a quantitative metric for spatial and temporal change detection;
(c) to evaluate the effectiveness of CDI maps in identifying both landscape degradation and signs of recovery in war-affected and depopulated areas.

## Materials and methods

### Study area

**Fig 1:**
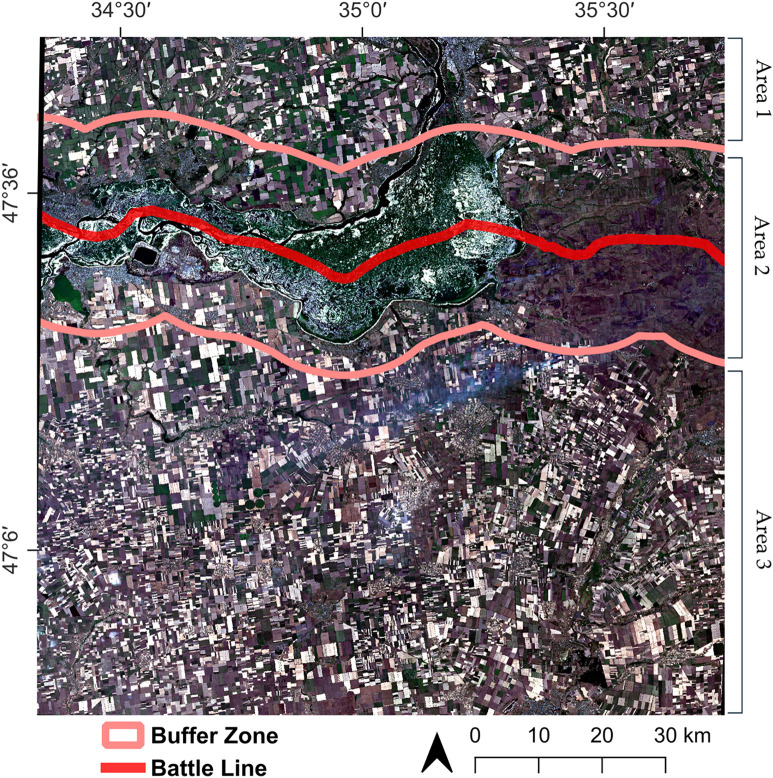
Location of the study area on the map of Ukraine and RGB composite of Sentinel-2 image from 14 July 2024. Basemap: Here Wego Terrain.

The study area of 10,000 km^2^ is located in southern Ukraine and includes parts of the Zaporizhzhia, Kherson, and Dnipropetrovsk Oblasts (Fig 1). It is traversed by a battle line that, as of April 2025, runs along the banks of the Kakhovka Reservoir, which was destroyed on 6 June 2023, and along the Dnipro River [40,41]. For ease of visual perception, the battle line is drawn through the geographic center of the Reservoir. About 84% of the study area is agricultural land, some of which has been abandoned due to depopulation – it has not been cultivated for at least two consecutive years, starting in the summer of 2022 [42].

The study area is divided into three areas (Fig 1). Area 1 includes the Ukrainian-controlled territories; Area 2 is a 15 km buffer zone on both sides of the battle line, within which organized agricultural activity is challenging; Area 3 includes the territories not controlled by Ukraine (as of April 2025). The width of Area 2, 15 × 2 km, was chosen for the following reason. The main weapons used in the Russo-Ukrainian war are numerous First Person View (FPV) drones, most of which have a range of up to 15 km (as of summer 2024) and, in rare cases, reach 20 km [43].

Table 1 provides a summary of the information for the three zones of the study area.

**Table 1:** Summary information on Area 1, Area 2. And Area 3 (14 July 2024).

Study Area	Area (km^2^)	Center coordinates (Lat, Lon)	Status
Area 1	1930	47.74680725, 35.08528011	Not occupied
Area 2	3455	47.52698051, 35.08353553	Partially occupied
Area 3	6535	47.12255075, 35.07306803	Occupied

### Data

The input data consists of Sentinel-2 L2A images at a spatial resolution of 10 meters, acquired in June–July 2020–2024. For each season, a scene with the lowest cloud cover was selected. Satellite images were acquired during the peak growing season (June–July) [44]. Each scene's spatial coverage corresponds to the location of the study area. Acquisition dates: 5 July 2020, 15 July 2021, 30 July 2022, 5 June 2023, 14 July 2024; sensing time: 08:35 UTC. Sensing parameters: relative orbit 064, tile and grid T36TXT. The Level-2A product processing level represents atmospherically corrected surface reflectance [45].

Satellite images are provided in open access by the Copernicus Browser (https://browser.dataspace.copernicus.eu). The location of the battle line was obtained from the Live Universal Awareness Map Liveuamap (https://liveuamap.com).

The computations were implemented using GIS RAPID 3.2 software [46].

Sentinel-2 Level-2A atmospherically corrected surface reflectance satellite data were used in this study. Prior to analysis, cloud-covered areas and cloud shadows were identified and excluded using preprocessing tools implemented in the RAPID GIS environment.

### Experimental design and validation

The study design ensures the comparability of multi-temporal satellite observations and enables the assessment of structural transformations in agricultural landscapes under varying intensities of anthropogenic pressure.

A consistent temporal framework was established by selecting one Sentinel-2 scene per year (2020–2024) during the peak growing season (June–July). This window was used to maximize the structural contrast between actively managed croplands and abandoned fields undergoing natural succession, while minimizing the influence of short-term intra-seasonal vegetation fluctuations. To examine how CDI values change across contrasting socio-economic and environmental contexts, the study area was stratified into three distinct zones based on the level of anthropogenic disruption: (i) government-controlled areas (stable management), (ii) a buffer zone along the frontline (high disturbance), and (iii) occupied territories (altered management).

A relative radiometric normalization procedure was applied to improve the comparability of CDI values across different atmospheric and illumination conditions. Spectrally stable areas identified through correlation analysis (r > 0.95) were used to derive a linear correction coefficient, allowing CDI values from different dates to be adjusted to a common reference level and reducing the influence of sensor and atmospheric variability on the observed changes.

Due to restricted access to the study area during active military operations, direct in-situ validation was not feasible. For this reason, an indirect validation strategy was adopted, based on comparison with regional-scale independent data, including official statistics on cultivated areas, population migration trends, and proxies of economic activity. While this approach does not provide pixel-level accuracy assessment, it supports the consistency of the CDI indicator at the regional scale and its alignment with documented socio-economic changes.

### Methods

This study examines the correspondence between the density of contours in Sentinel-2 satellite images and the condition of agricultural landscapes. A contour is an extended zone of a sharp gradient in the Sentinel-2 reflectance in one of the regions of the electromagnetic spectrum (bands) [47]. The contour density in a given area (sliding window) is the sum of the reflectance gradient modules along the selected contours divided by the window area.

Assume that the state of the landscape does not affect the contour density in satellite images. Consequently, its changes, including those caused by external factors, do not lead to systematic changes in contour density.

To refute this assumption, suppose that the contour density in Sentinel-2 images corresponds to changes in the state of agricultural landscapes. In particular, a decrease in anthropogenic impact, such as the suspension of agricultural activities in war zones, leads to a decrease in contour density.

In a narrower sense, the degradation (or abandonment) of agricultural landscapes results in a decrease in the density of landscape elements due to the loss of anthropogenic structures.

A quantitative indicator is proposed – the Contour Density Indicator, which measures the average number and contrast of contours on Sentinel-2 satellite images at each imaging moment. The difference of CDI maps (ΔCDI) for two Sentinel-2 scenes allows the analysis of changes between two points in time.

The Canny algorithm, which is highly accurate and noise-resistant, is used to extract contours [48,49]. Canny algorithm consists of six sequential steps [50]:

1. Convert the image to greyscale.
2. Remove noise from the image using a Gaussian filter (Gaussian smoothing kernel – 1.0).
3. Apply a Sobel (upper threshold – 0.9, lower threshold – 0.5), Prewitt, or other gradient filters to calculate the magnitude and direction of the gradient.
4. Suppress non-maximum values.
5. Edge thinning.
6. Hysteresis thresholding to detect and suppress “weak” edges that are not connected to “strong” edges.

**Fig 2:**
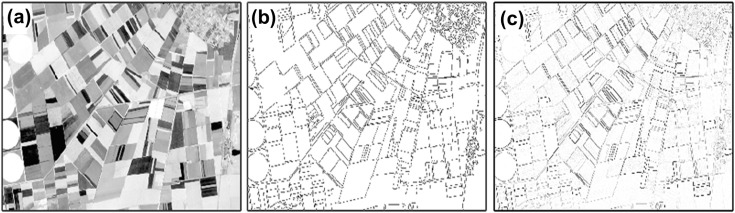
Examples of contour mapping: (a) fragment of the study area in Sentinel-2 band 4 (B04) from 5 July 2020; (b) Canny algorithm; (c) modified Canny algorithm.

**Fig 3:**
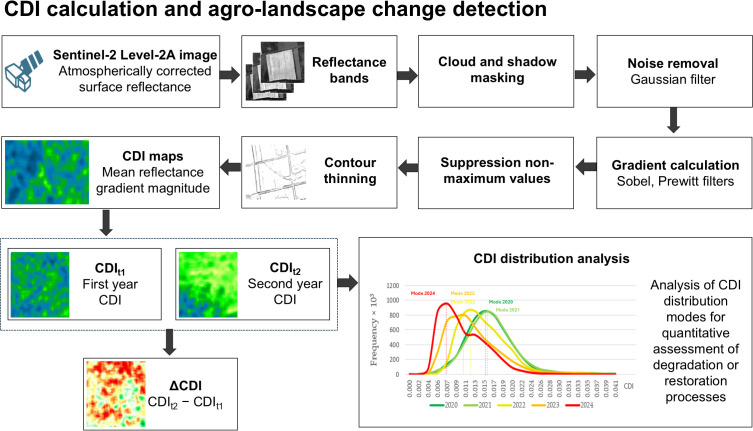
General calculation scheme.

However, in the proposed approach, the final binarization step of the Canny algorithm is omitted to preserve information about the gradient amplitude, which characterizes the “strength” of contours and is important for assessing their degradation as a result of reduced anthropogenic impact in conflict zones (Fig 2) [50,51]. The algorithm was applied to Sentinel-2 images with pre-enhanced contrast (Fig 3). The resulting image contains refined and noise-cleaned contours, represented at pixels with coordinates *(i,j)* by gradient values *G*_*i,j*_. The gradient amplitude characterizes the contrast of the contour. If there is no contour, the gradient value is zero.

The CDI is calculated as the average gradient value in a sliding window of size *M × N* for each pixel of the image:


$${CDI}_{i,j}=\frac{1}{M\times \text{N}}\sum_{m=\frac{-M}{2}}^{\frac{M}{2}}\sum_{n=-\frac{N}{2}}^{\frac{N}{2}}G_{i+m,j+n}$$ (1)

CDI measures the density and intensity of contours within a window. The difference between CDI maps (ΔCDI) obtained from images taken before (*t*_*1*_) and after (*t*_*2*_) the onset of depopulation processes allows changes in contours to be identified and the destructive impact to be assessed. Additional information can be obtained from the histograms of the CDI value distributions (Fig 3).


$$\begin{array}{c} \Delta CDI=\;{CDI}_{t2}-\;{CDI}_{t1} \end{array}$$ (2)

**Fig 4:**
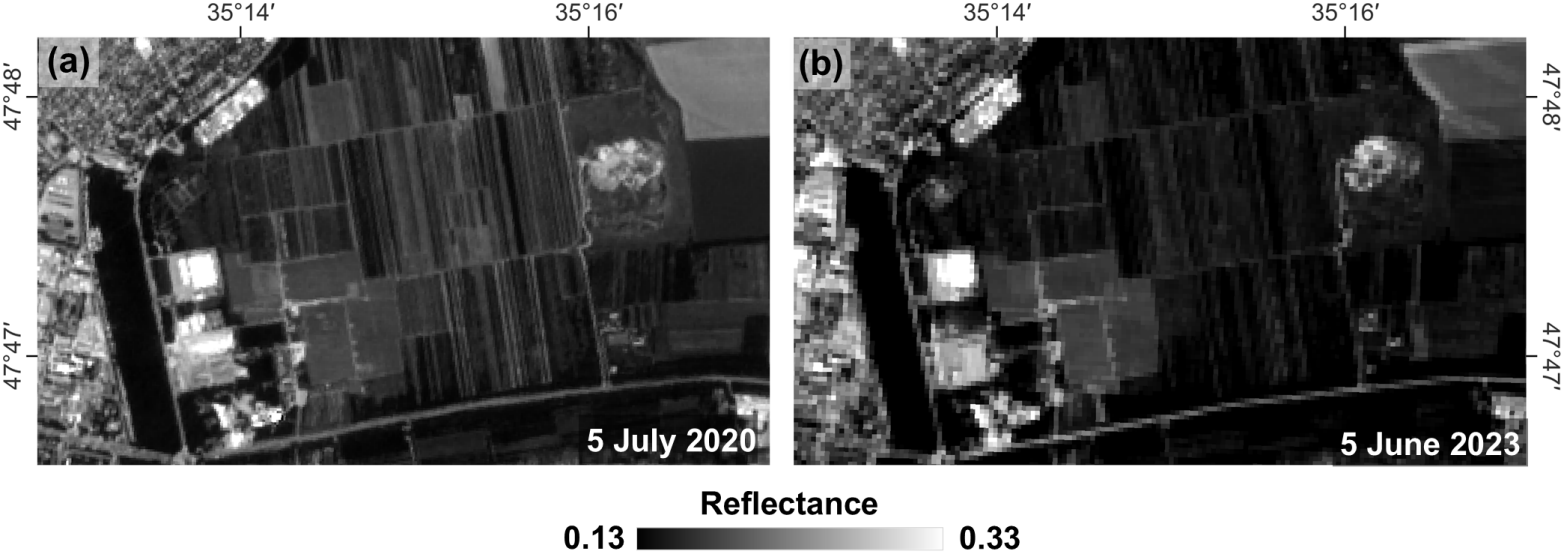
Example of contour sharpness distinction on multi-temporal Sentinel-2 B04 images for the Zaporizhzhia city fragment on (a) 5 July 2020 and (b) 5 June 2023.

Computational experiments have revealed that CDI values depend on the sharpness and brightness of the original images, which are particularly affected by the weather conditions at the time of acquisition. Fig 4 shows a fragment of agricultural land near Zaporizhzhia city in two images taken on two different dates: differences in contour sharpness led to considerable variations in CDI values, although they should be minimal.

To ensure comparability between images acquired under different atmospheric and illumination conditions, a correction factor k was introduced. This factor is estimated using spectrally stable areas identified through correlation analysis (*r* > 0.95) between multi-temporal CDI maps.

Within these “stable” areas, the mean CDI values for the first $\left(CD\overset{\left(1\right)}{\underset{avg}{I}}\right)$ and second $\left(CD\overset{\left(2\right)}{\underset{avg}{I}}\right)$ images are calculated. The correction factor is then applied to adjust CDI values of the second image:


$$\begin{array}{c} \overset{\left(2^{\prime }\right)}{\underset{i,j}{CDI}}=k\times \overset{\left(2\right)}{\underset{i,j}{CDI}} \end{array}$$ (3)

Correcting coefficient *k* is calculated as follows:


$$\begin{array}{c} k=\frac{\overset{\left(1\right)}{\underset{avg}{CDI}}}{\overset{\left(2\right)}{\underset{avg}{CDI}}} \end{array}$$ (4)

The corrected CDI values ensure proper comparison of the two CDI time maps and the generation of a change map.

Normalized difference (dmCDI) of CDI mode in *t*_*2*_ year ($\overset{\left(t2\right)}{\underset{mode}{CDI}}$) relative to CDI mode in 2020 ($\overset{\left(2020\right)}{\underset{mode}{CDI}}$) was additionally calculated:


$$\begin{array}{c} dmCDI\;=\;\frac{\overset{\left(t2\right)}{\underset{mode}{CDI}}-\overset{\left(2020\right)}{\underset{mode}{CDI}}}{\overset{\left(2020\right)}{\underset{mode}{CDI}}}\; \end{array}$$ (5)

The dmCDI value shows the relative change in CDI mode, allowing comparison of mode change dynamics between different areas or time periods, regardless of the initial CDI level. Positive dmCDI values indicate an increase in contour density (landscape recultivation), negative dmCDI values indicate a decrease in contour density (landscape degradation), and dmCDI values close to zero indicate stability in the spatial structure of the landscape.

## Results

The methodology described above was used to generate CDI maps for the study area. The time interval considered was 2020–2024, represented by five summer Sentinel-2 images.

The B04 (red) band of the Sentinel-2 L2A image was processed. B04 was chosen for several reasons: 1) it has a high spatial resolution of 10 m, unlike several others; 2) the red band allows the study of both vegetation and soil, as well as urbanized areas; 3) empirical data allowed to conclude that CDI maps obtained from this band are the most sensitive to changes in contour density over time [50,52]. Sensitivity analysis across Sentinel-2 spectral bands revealed that Band 4 (B04) yields the highest CDI sensitivity, exhibiting the largest shift in mode values, while Bands 3 (B03) and, to a lesser extent, Band 2 (B02) demonstrate comparable but weaker responses [50].

**Fig 5:**
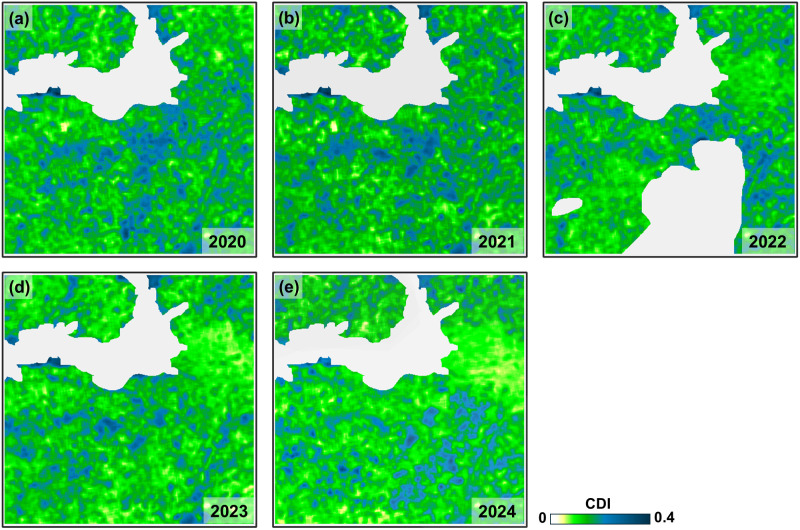
CDI maps of the study area based on Sentinel-2 B04 data on (a) 5 July 2020; (b) 15 July 2021; (c) 30 July 2022; (d) 5 June 2023; (e) 14 July 2024.

Fig 5 shows the resulting CDI maps, representing the average contour density in a 2 × 2 km sliding window. Presumably, in anthropogenically altered agricultural regions, a substantial proportion of the detected contours are directly related to human activity, so contour density should change regularly with depopulation and decline in agricultural activity. Conversely, population recovery and agricultural production expansion should be followed by an increase in contour density.

The maps in Fig 5 confirm this assumption and demonstrate the complex nature of CDI dynamics over time: in peacetime, in 2020–2021, there is a slight increase in the index overall, followed by a sharp decline during the period of hostilities from 2022 to 2024, and in 2023–2024, except the area along the battle line, there is a partial recovery of CDI values, especially in Area 1.

**Fig 6:**
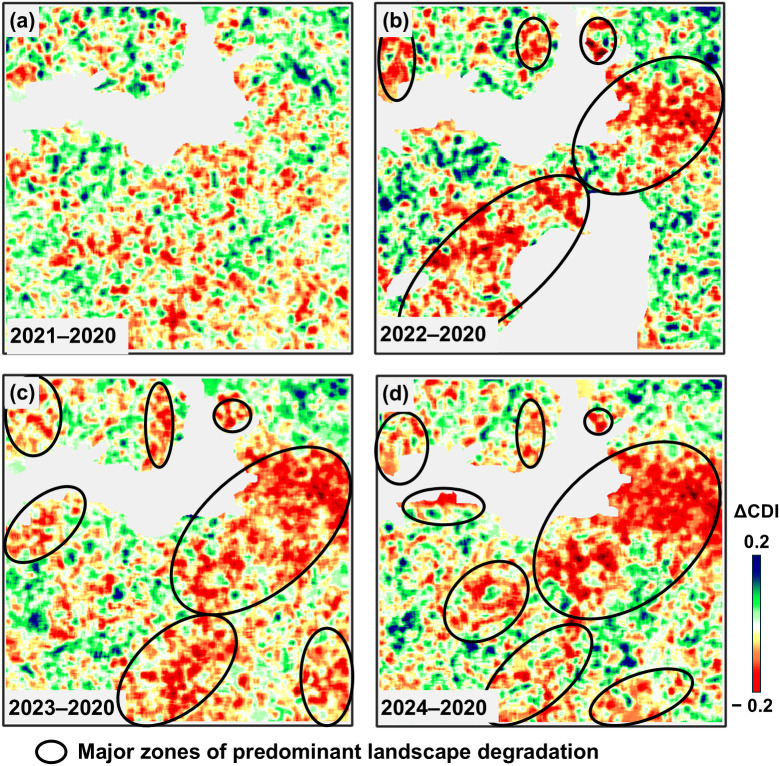
ΔCDI maps for 2021–2024 relative to 2020: (a) 2021–2020; (b) 2022–2020; (c) 2023–2020; (d) 2024–2020.

Fig 6, which shows ΔCDI maps for 2021–2024 relative to 2020, confirms the trends observed in the analysis of CDI maps for 2020–2024. During periods without military action, the changes are local and multidirectional (Fig 6a). However, with the start of the full-scale war, the negative trends in land use intensify and are accompanied by the emergence of large areas with sharp declines in ΔCDI (Fig 6b–6d). In 2024, there is some recovery in population and agricultural activity in areas outside Area 2, leading to a corresponding increase in ΔCDI values in Area 1 and Area 3. The ellipses in Fig 6 show the main zones of the study area with predominantly negative ΔCDI values, where a decrease in contour density is observed, indicating agricultural landscape degradation processes.

Table 2 shows the mode values of 2020–2024 CDI maps (Fig 5) for the entire study area and individually for Areas 1, 2, and 3.

**Table 2:** CDI mode values.

Year	Area 1		Area 2		Area 3		Study Area	
	CDI_mode_	dmCDI	CDI_mode_	dmCDI	CDI_mode_	dmCDI	CDI_mode_	dmCDI
**2020**	0.0138		0.0162		0.0157		0.0157	
**2021**	0.0150	0.085	0.0167	0.034	0.0155	−0.017	0.0161	0.021
**2022**	0.0143	0.032	0.0126	−0.220	0.0143	−0.095	0.0151	−0.042
**2023**	0.0117	−0.150	0.0099	−0.386	0.0133	−0.152	0.0137	−0.128
**2024**	0.0132	−0.046	0.0073	−0.547	0.0132	−0.164	0.0128	−0.187

**Fig 7:**
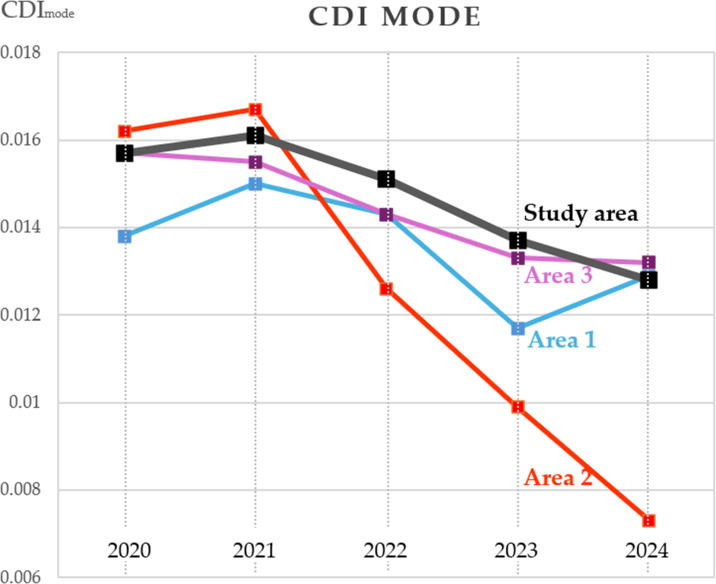
Plots of 2020–2024 CDI mode changes over time for the entire study area and Areas 1–3.

Based on the data in Table 2, plots were generated in Fig 7 showing the dynamics of CDI modal values in 2020–2024 for the entire study area and Areas 1–3.

The mode values obtained for the entire study, after a slight increase in 2021, have steadily decreased since the beginning of depopulation caused by the war, which corresponds to a decrease in contour density.

More detailed information can be obtained by analyzing the changes in the mode values of the CDI for individual areas.

The values for Area 1, corresponding to the Ukraine-controlled part of the territory, indicate a positive dynamic in the contour density in 2024, which can be attributed to the partial return of the residential population and the corresponding increase in economic activity.

Area 2, corresponding to a 30-kilometer-wide zone along the battle line, became the site of continuous combat operations in 2022–2024 and shows a steady decline in modal CDI values. This zone, often called the “kill zone,” is entirely unsuitable for organized agricultural activity, as evidenced by a sharp decline in the density of object contours on satellite images.

Area 3, representing the occupied territory, similarly to Area 1, shows signs of recovery in 2024, as evidenced by a slowdown in the decline rate of the CDI mode. However, unlike Area 1, the mode does not grow but continues to decrease.

**Fig 8:**
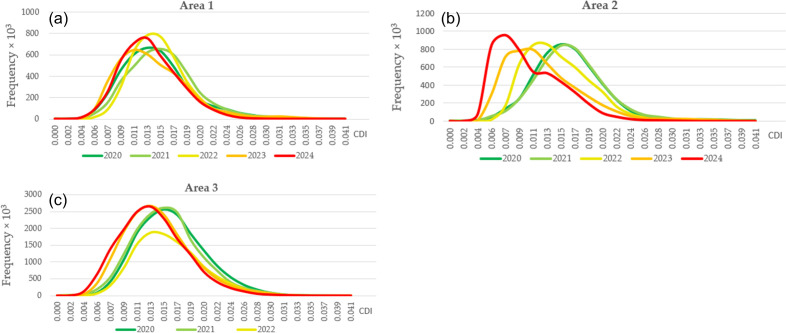
Histograms of CDI value distribution: **(a)** Area 1; **(b)** Area 2; **(c)** Area 3.

These conclusions are confirmed by histograms of the CDI values for Areas 1–3 (Fig 8).

The plots in Fig 8 show the trends previously observed in Figs 5 and 6. In peacetime, the contour density remains stable or varies slightly. Accordingly, the mode values of the CDI in 2020 and 2021 are almost equal, and the shape of the histograms is very similar. However, in 2022, with the onset of war and depopulation, the contour density began to decrease, reflecting a decrease in the mode of all histograms for 2022 and 2023 in all three areas. In 2024, the mode continues to decrease in Areas 2 and 3, corresponding to the further degradation of agriculture, while it slightly increases in Area 1, which can be attributed to the regrowth of the population in the previously unoccupied territory of Ukraine.

## Discussion

The results of this study demonstrate that contour-based metrics derived from Sentinel-2 imagery can capture structural changes in agricultural landscapes under conditions of reduced anthropogenic activity. The observed decline in the Contour Density Indicator (CDI) across the study period, particularly within the battle-line buffer zone, provides evidence that the degradation of agricultural systems is associated with a measurable loss of spatial structure.

The suspension or reduction of agricultural activities is associated with a decrease in contour density in the images. At the same time, during periods when economic activity is not affected by destructive events, contour density generally remains stable or varies randomly without a trend (Fig 5a, 5b). This conclusion is most clearly confirmed by the shift in CDI mode towards lower values during active hostilities (2022–2023), while during peacetime (2020–2021) or when the population partially returns to their former areas of residence (2023–2024, Areas 1 and 3), contour density remains stable or even increases (Table 2, Figs 7–8).

However, this important conclusion needs to be quantified: what is the nature and strength of the relationship between the intensity and duration of military operations and contour density? To answer this question, the following computational experiment was conducted.

### Assessment of the type and strength of the relationship between anthropogenic impact on the agricultural landscape and contour density

**Fig 9:**
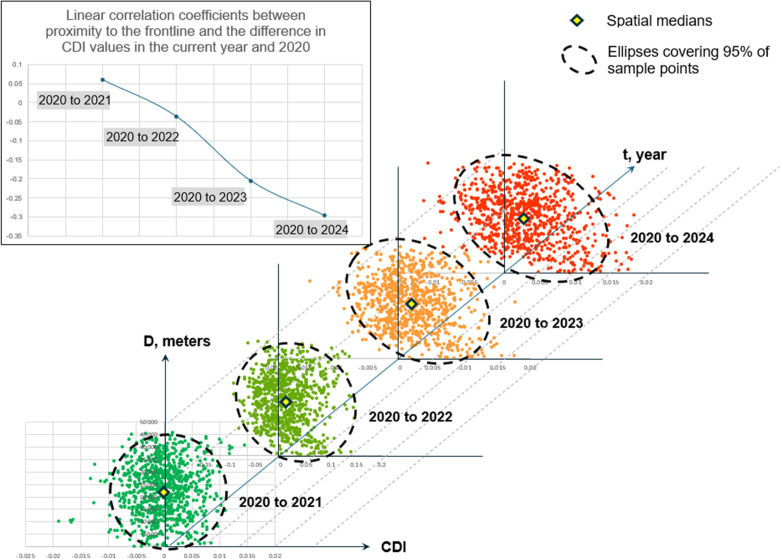
The type and strength of the relationship between proximity to the battle line and changes in CDI.

In the study area, 1000 points were randomly selected, each assigned two indicators: distance to the battle line (Fig 1) and the difference between the CDI values for the current year and 2020. It is assumed that the closer a point is to the battle line and the longer the hostilities continue, the stronger the effect of contour density reduction will be. The plot showing the distribution of 1000 points according to the specified characteristics is shown in Fig 9.

In pre-war 2021, there was no correlation between the examined characteristics, as noted earlier, because, in the absence of catastrophic events such as war, CDI values generally change without a clear pattern.

The military actions that began in early 2022 resulted in a rightward shift of the spatial median, indicating a decrease in CDI values relative to 2020. A very weak negative correlation between the two parameters began to appear.

2023 was the most active period of military operations in the study area, with a stable battle line. The negative correlation coefficient reached −0.2, and the spatial median shifted even more.

In 2024, hostilities continued, although they were less intense than in 2023. The growth of the negative correlation between proximity to the battle line and the difference in CDI slowed down compared to 2020, reaching a value of −0.3.

Although the correlation achieved is weak, its steady increase over time is noteworthy. It can be assumed that the correlation will increase if future military operations continue.

Thus, it can be concluded that the relationship between the condition of agricultural landscapes and contour density is complex and determined by various social and natural factors. Based on the available data, a linear relationship can be assumed. Its strength is directly related to the duration of the impact of the factors causing changes in the agricultural landscape.

### Validity Assessment

Validating the in situ results is a complex challenge. Since the study area is a war zone and part of it is under foreign occupation, field research is either extremely difficult or impossible. For the same reasons, as well as for confidentiality reasons, statistical data are limited and imprecise.

Consequently, the few indirect data available were used to assess validity. However, all of them showed satisfactory agreement with the results obtained.

**Fig 10:**
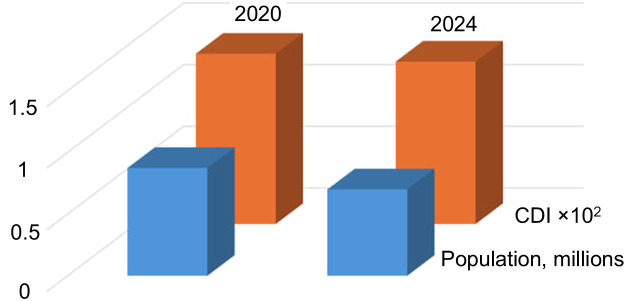
CDI mode and population of the Zaporizhzhia district in 2020 and 2024 (according to the Ukrainian Institute of Demography).

**Fig 11:**
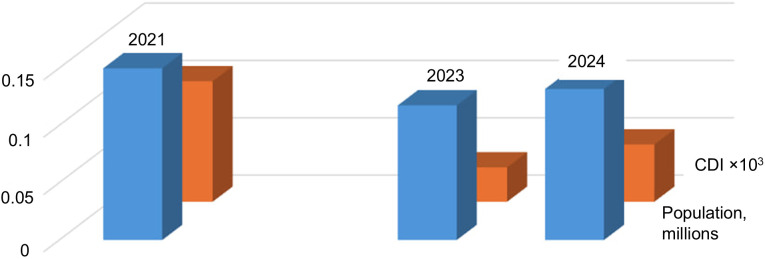
CDI mode and population of Nikopol in 2021–2024 (data from the Institute of Demography of Ukraine (2021) and municipal estimates (2023–2024)).

Depopulation was assumed to be the most crucial factor in reducing the number of contours in the images. Figs 10 and 11 show population estimates for the Zaporizhzhia district (right part of Area 1) and Nikopol city (the largest settlement in the left part of Area 1). For comparison, the modal CDI values for Area 1 are shown.

Both plots demonstrate a consistent change in CDI mode and population size, confirming previously identified trends: changes in the number of contours are directly related to changes in population size in agricultural regions. It should be noted that in the plots, the change in the number of contours occurs without a considerable time lag, following the population. However, additional data is needed for a more accurate assessment of the mutual dynamics of these indicators.

**Fig 12:**
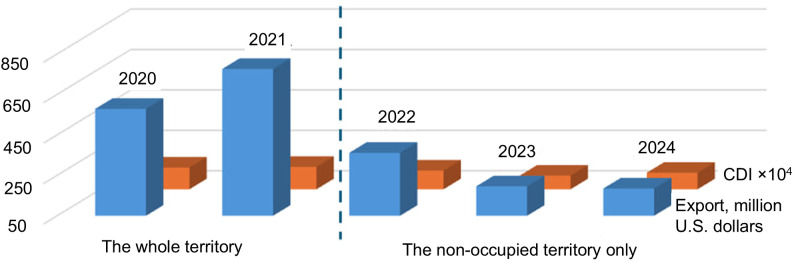
CDI mode and agricultural exports from the Zaporizhzhia Oblast in 2020–2024 (data from the Main Department of Statistics in the Zaporizhzhia Oblast, zp.ukrstat.gov.ua).

A more important indicator directly related to the state of agricultural landscapes is the export of agricultural products from the study area. Fig 12 shows data for the non-occupied part of the Zaporizhzhia Oblast (right part of Area 1).

When analyzing the plot in Fig 12, it should be noted that the data for 2020 and 2021 are given for the entire territory of the Oblast, while the data for 2020–2024 are given only for the northern part of the Oblast, which is under Ukrainian control. Within these two periods, there is also a consistent change in the indicators. Similar results were obtained for several other social and economic indicators, particularly the number of registered entrepreneurs, imports of goods to Zaporizhzhia Oblast, and others.

Figs 10–12 generally confirm the validity of the results, although this confirmation is based on a limited set of currently available data.

### Sentinel-2 bands selection for CDI calculation

In this study, band 4 of the Sentinel-2 image was used to calculate the CDI, but other bands were also tested. The best results, ensuring the sensitivity of the CDI to landscape changes, were obtained using bands 2, 3, 4, 5, and 11. At the same time, maps based on bands 3 and 4 provide the widest range of values, allowing good territory differentiation and selection of any of them for CDI calculations.

**Fig 13:**
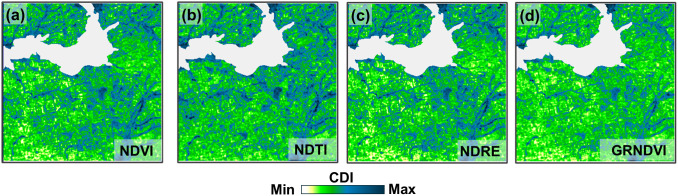
Example of CDI maps calculated using NDVI, NDTI, NDRE, and GRNDVI (Sentinel-2 image acquisition date: 14 July 2024).

In addition, an attempt was made to calculate CDI based on spectral indices rather than individual Sentinel-2 bands. The potential of NDVI, Normalized Difference Tillage Index (NDTI), Normalized Difference Red Edge Index (NDRE), and Green-Red NDVI (GRNDVI) was investigated [53–55]. The CDI maps constructed on their basis showed excessive sensitivity to hydrographic objects, against which the contours of other types of objects (such as cropland boundaries) are poorly visible (Fig 13), while the strip along the battle line (Area 2) is almost indistinguishable.

The unfavorable assessment of the prospects for using these specific indices does not negate the possibility of further searching for combinations of bands and vegetation indices that could provide CDI maps more suitable for delineating the contours of spatial objects.

### The effect of sliding window size

CDI maps were generated in a sliding window that summarizes the data on the contours within it. In this study, a 2 × 2 km window was used. Thus, CDI generalizes information based on the contours of individual objects over large areas. Unlike traditional methods of detecting temporal changes, CDI does not track specific physical objects, which can be interpreted as a disadvantage.

It should be noted, however, that it is possible to compute CDI with a smaller window size, e.g., 3 × 3 pixels, allowing CDI to be analyzed with the same level of detail as the original image. However, using a large window offers several important advantages. The most important is the clarity and visibility of the resulting image. By eliminating the small details that result from generalizing contours, it is possible to focus on the essential features and imbalances in the area's development, ignoring random anomalies.

**Fig 14:**
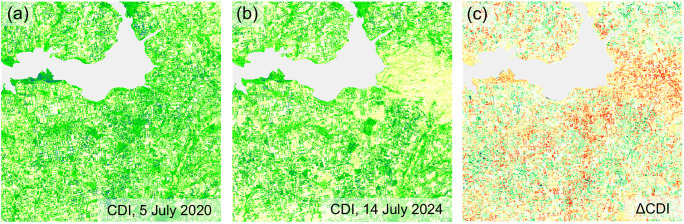
CDI (a, b) and ΔCDI (c) maps generated in a 200 × 200 m sliding window using the B04 of Sentinel-2 images from 5 July 2020, and 14 July 2024.

This is especially important when making management decisions at the level of large territorial units. Otherwise, the CDI can be calculated in a small window, providing useful data at the local level. For example, Fig 14 shows CDI maps obtained in a sliding window size of 200 × 200 meters.

These maps are much more detailed than those shown in Figs 5 and 6. Although analyzing them requires considerable effort, this can be justified when studying the area in detail.

Thus, the choice of window size when calculating the CDI should depend on the scale of the study and the nature of the tasks to be solved.

### Comparative assessment of the proposed methodology

The problem of finding temporal differences in satellite imagery arose with the advent of satellite imagery itself. This is one of the most obvious and straightforward applications of remote sensing. Accordingly, many methods and approaches exist to detect and monitor image changes, some of which are described in [56–59].

The approach proposed in this study is not considered as a replacement for existing ones, although it has several clear advantages over the basic approaches.

For example, when compared to methods based on the use of NDVI vegetation index maps in the study area, the following advantages of CDI maps can be noted: CDI is highly sensitive to decreases in agricultural activity compared to NDVI; CDI has lower sensitivity to short-term changes in vegetation conditions.

**Fig 15:**
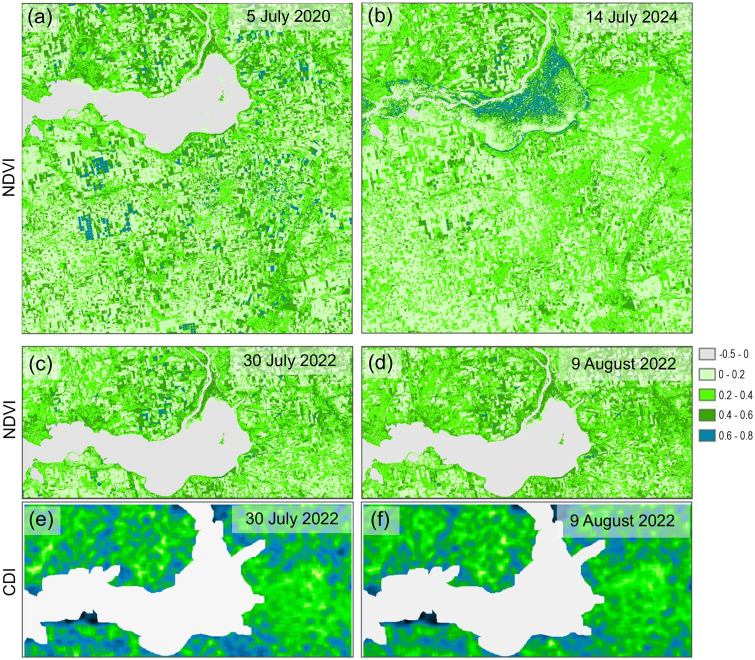
Comparison of NDVI and CDI maps illustrating vegetation dynamics and land degradation in the study area. NDVI maps for (a) 5 July 2020 and (b) 14 July 2024; NDVI (c, d) and CDI (e, f) maps for two close dates: (c, e) 30 July 2022 and (d, f) 9 August 2022.

These statements are illustrated in Fig 15. The NDVI maps derived from Sentinel-2 imagery for 5 July 2020 and 14 July 2024 are presented in Fig 15a and 15b, respectively. A general decline in vegetation cover across the entire area is clearly visible [50]. However, the strip along the former battle line, which experienced the most significant war-related damage and is distinctly visible on the CDI maps (Fig 5), is barely noticeable on the NDVI maps. Without additional data, identifying this zone based on NDVI alone would be extremely difficult.

Figs 15c–15f show selected areas of the CDI (Figs 15c and 15e) and NDVI (Figs 15d and 15f) maps corresponding to two dates in close succession – 30 July 2022 and 9 August 2022. Since these dates are only days apart and no significant military activity occurred in the area during this period, minimal differences would be expected between the maps. A side-by-side comparison reveals that the NDVI maps exhibit far more noticeable variations than the CDI maps. This observation is supported by numerical data: the mean change in CDI values was only 3.3%, whereas NDVI values fluctuated by 13.1%. For reference, the change in the B04 band of the original Sentinel-2 images for the same period was 10.9%. These findings highlight a key limitation of NDVI-based analysis – natural fluctuations in vegetation condition can mask or exaggerate actual land degradation signals, making NDVI unreliable for consistent monitoring, especially when comparing images from different dates within the same growing season or across different years. In contrast, CDI maps are far less influenced by seasonal dynamics or short-term environmental factors, allowing for a more stable and objective assessment of land degradation caused by armed conflict or other large-scale disruptive events.

Undoubtedly, the set of methods available to researchers for tracking changes is not limited to NDVI map analysis. Hundreds of vegetation indices and numerous algorithms can be applied to the original images [60–65]. This study did not aim to prove the advantages of the proposed methodology in a specific area of the Earth's surface at a particular time. A more important task was to investigate the possibility of using contour density as a parameter related to the population's economic activity in agriculture. Southern Ukraine, which has suffered from years of war, is a place where the harmful processes leading to the degradation of agricultural landscapes are clearly visible.

Although CDI and ΔCDI maps can be used directly to assess changes in the landscape, they can also provide supplementary input for other methods. In addition, conclusions and management decisions based on the analysis of CDI and ΔCDI maps can be refined and adjusted based on the results of other methods.

### Relationship between contour density and landscape transformation

The findings confirm the initial hypothesis that a reduction in agricultural activity leads to a decrease in contour density. This effect is especially pronounced in areas exposed to prolonged military activity, where the destruction of infrastructure, abandonment of fields, and cessation of land management practices result in the gradual disappearance of well-defined field boundaries. As shown in Table 2, the CDI mode decreased by up to 55% relative to 2020 in the most affected zones, indicating substantial structural simplification of the landscape.

At the same time, areas under stable control exhibited partial recovery of CDI values in 2024, suggesting that contour density is sensitive not only to degradation processes but also to the resumption of agricultural activity.

The analysis of the relationship between CDI change and distance to the battle line further supports this interpretation. Although the correlation remains moderate, its consistent increase over time suggests that the cumulative impact of anthropogenic disruption plays a critical role in shaping landscape structure. This indicates that contour density reflects not only instantaneous changes but also the duration and intensity of external disturbances.

### Comparison with existing approaches

Most existing approaches to monitoring agricultural abandonment and land-use change rely on spectral indices such as NDVI or on classification-based methods, including recent deep learning and semantic segmentation techniques applied to high-resolution satellite imagery (e.g., convolutional neural networks for field boundary extraction and land-use classification). While such approaches can achieve high accuracy, they typically require large labeled datasets, significant computational resources, and careful model generalization across regions. Thus, studies based on spectral indices often rely on empirical relationships and remain sensitive to atmospheric and seasonal variability [66]. Recent studies increasingly employ multi-source data and machine learning techniques, achieving high classification accuracy but requiring extensive training datasets and complex model calibration [67].

In contrast, the proposed contour-based approach focuses on the spatial configuration of landscape elements rather than their spectral properties to detect structural degradation even when land-use categories remain formally unchanged. For example, while NDVI-based methods may fail to distinguish between actively cultivated and recently abandoned fields during certain phenological stages, CDI captures the gradual loss of boundary definition and spatial heterogeneity.

The comparison between CDI and NDVI presented in this study demonstrates that CDI exhibits significantly lower temporal variability under stable conditions, making it more suitable for multi-temporal analysis in heterogeneous and dynamic environments. At the same time, the proposed method does not replace spectral or machine learning approaches but rather complements them by providing an interpretable, training-free indicator of landscape structure.

### Practical and theoretical implications

From a practical perspective, the proposed methodology offers a valuable tool for monitoring agricultural regions where ground-based observations are limited or unavailable. This is particularly relevant in conflict zones, disaster-affected areas, or regions with restricted access. The ability to detect structural degradation without requiring prior land-use classification or training data significantly reduces the complexity of large-scale monitoring.

In addition, CDI provides a quantitative measure of landscape “abandonment intensity,” which can support damage assessment and recovery planning by governmental agencies and international organizations (e.g., in the context of food security monitoring or post-conflict land management). Such a metric may also be useful for comparative analysis across regions and time periods where consistent ground data are lacking.

From a theoretical standpoint, the results can be interpreted within the framework of landscape ecology and land-use change theory. The reduction in CDI reflects a transition from a highly organized, anthropogenically structured (“technogenic”) landscape toward a more heterogeneous and weakly structured state driven by natural succession processes. This interpretation is consistent with theories of landscape fragmentation and edge dynamics, where the loss of managed boundaries leads to increased spatial homogenization and reduced structural complexity. In this sense, CDI can be viewed as a proxy indicator of the degree of anthropogenic organization of agricultural space.

### Limitations

Despite its advantages, the proposed approach has several limitations.

The lack of in-situ verification due to security constraints in the study area limits the ability to perform direct validation. As a result, validation relied on indirect indicators such as statistical data on cultivated areas, population dynamics, and economic activity. While these sources provide supporting evidence, they do not fully substitute for ground-based measurements.

Method depends on image quality and preprocessing conditions. Variations in illumination, atmospheric effects, and sensor characteristics can influence contour detection and CDI values, although the introduced correction procedure partially mitigates these effects.

The use of a single spectral band (B04) may limit the sensitivity of the method to certain types of landscape changes. Although empirical analysis showed that this band provides high sensitivity to contour structures, the integration of multispectral information or adaptive band selection could further improve robustness.

CDI is calculated using a sliding window approach, and the choice of window size affects the level of spatial generalization. Window size can be arbitrarily small (e.g., on the order of the native pixel resolution of 10 × 10 m), allowing highly localized analysis. However, in this study, a 2 × 2 km window was selected to emphasize the most significant patterns of landscape degradation while maintaining visual correspondence with major landscape elements such as settlements, forest patches, and large agricultural fields. This choice represents a trade-off between noise reduction, interpretability, and spatial detail. Smaller windows would increase sensitivity to local variability but reduce the clarity of regional patterns.

### Future research directions

Future work should focus on improving validation strategies by incorporating additional independent data sources, such as high-resolution imagery, or field surveys (where feasible). The integration of CDI with other indicators, including spectral indices and machine learning-based classification methods, may also enhance the overall accuracy and interpretability of change detection.

Another promising direction is the extension of the proposed methodology to other regions and types of disturbances, such as natural disasters, climate-driven land degradation, or socio-economic transitions. This would allow the generalizability of the CDI concept to be assessed across different environmental and geographical contexts.

In addition, further research is needed to investigate the optimal parameterization of the contour detection algorithm and the potential benefits of incorporating multi-band or multi-temporal features directly into the CDI calculation.

Overall, the results demonstrate that contour density is a meaningful and sensitive indicator of structural changes in agricultural landscapes. The proposed CDI-based approach provides a complementary perspective to existing remote sensing methods and shows strong potential for application in conditions where traditional monitoring techniques are limited.

## Conclusions

The density of object contours identified in Sentinel-2 satellite images can be used as an indicator of agricultural landscape condition. Accordingly, studying the dynamics of contour changes allows assessing the processes of degradation/reclamation of agricultural landscapes occurring in response to changes in nature and intensity of economic activity.

For example, depopulation in southern Ukraine caused by prolonged warfare has resulted in the abandonment of some cultivated agricultural areas and the collapse of related infrastructure. These processes have led to a decrease in contour density on Sentinel-2 satellite images.

The Contour Density Indicator, based on the number and contrast of boundaries in an image, enabled the quantification of changes in the spatial structure of agricultural areas in southern Ukraine between 2020 and 2024. This is clearly shown in CDI maps and their differences for 2020–2024 (2.5 years before and 2.5 years after the start of the war). The maps provide both areas of degradation of agricultural landscape contours, particularly evident in a 30 km wide zone along the battle line, and areas of partial restoration of agricultural activity.

An analysis of images taken over a five-year period revealed that during peacetime (2020–2021), the density of contours remained stable. However, with the onset of active hostilities (2022–2023), a systematic decrease was observed, particularly in the battle line zone. The partial return of the residential population to Ukrainian-controlled areas in 2024 led to some recovery of the CDI values.

Thus, the results demonstrate the fundamental possibility of using information on the state of landscape contours to assess the dynamics of its degradation/recultivation and are consistent with state statistics on population change and agricultural exports from the affected territory. CDI and ΔCDI maps can be used to rapidly monitor agricultural landscape degradation in conditions of strictly limited access to field measurements.

Further research includes evaluating the potential use of higher and lower resolution images for CDI calculation, searching for vegetation indices and band combinations that provide advantages over the use of B04 for CDI calculation, developing AI methods to eliminate the influence of non-agricultural object contours (fortifications, military logistics facilities, etc.), and selecting and testing methods for integrating CDI with other data.

## References

[1] Halkin V. The Role of Ukraine in Ensuring Global Food Security: Current Challenges and Prospects. GJNR. 2024;7(3):s396–419. doi: 10.33002/nr2581.6853.0703ukr20

[2] Arreyndip NA. The Russia–Ukraine Conflict: A Global Impact Assessment in the Corn and Wheat Sectors. Agriculture. 2025;15(5):550. doi: 10.3390/agriculture15050550

[3] Dai K, Cheng C, Kan S, Li Y, Liu K, Wu X. Impact of Arable Land Abandonment on Crop Production Losses in Ukraine During the Armed Conflict. Remote Sensing. 2024;16(22):4207. doi: 10.3390/rs16224207

[4] Food and Agriculture Organization. The importance of Ukraine and the Russian Federation for global agricultural markets and the risks associated with the war in Ukraine. Rome: FAO. 2022. https://openknowledge.fao.org/server/api/core/bitstreams/3d62caef-1749-404e-8217-6ac4783a135b/content

[5] Chen B, Tu Y, An J, Wu S, Lin C, Gong P. Quantification of losses in agriculture production in eastern Ukraine due to the Russia-Ukraine war. Commun Earth Environ. 2024;5(1). doi: 10.1038/s43247-024-01488-3

[6] Nasibov A, Shebanina O, Kormyshkin I, Gamayunova V, Chernova A. The impact of war on the fields of Ukraine. Int J Environ Stud. 2024;81:159–68. doi: 10.1080/00207233.2024.2314889

[7] Rexhepi BR, Berisha BI, Xhaferi BS. Analysis of the impact of the war on the economic state of agriculture in Ukraine. Econ Aff. 2023;68:839–44. doi: 10.46852/0424-2513.2S.2023.29

[8] Peterson S, Husak G, Shukla S, McNally A. Crop area change in the context of civil war in Tigray, Ethiopia. Environ Res: Food Syst. 2024;1(1):015003. doi: 10.1088/2976-601x/ad3559

[9] Holail S, Saleh T, Xiao X, Xiao J, Xia G-S, Shao Z, et al. Time-series satellite remote sensing reveals gradually increasing war damage in the Gaza Strip. Natl Sci Rev. 2024;11(9):nwae304. doi: 10.1093/nsr/nwae304 39309412 PMC11413530

[10] Cherevko H. Challenges for the agriculture of Ukraine during the war and directions of its development. Annals PAAAE. 2024;XXVI(1):43–55. doi: 10.5604/01.3001.0054.2828

[11] Ma Y, Lyu D, Sun K, Li S, Zhu B, Zhao R, et al. Spatiotemporal Analysis and War Impact Assessment of Agricultural Land in Ukraine Using RS and GIS Technology. Land. 2022;11(10):1810. doi: 10.3390/land11101810

[12] Li X, Ma L, Liu X. Identification, Mechanism and Countermeasures of Cropland Abandonment in Northeast Guangdong Province. Land. 2025;14(2):246. doi: 10.3390/land14020246

[13] Hnatushenko VV, Sierikova KYu, Sierikov IYu. Development of a Cloud-Based Web Geospatial Information System for Agricultural Monitoring Using Sentinel-2 Data. In: 2018 IEEE 13th International Scientific and Technical Conference on Computer Sciences and Information Technologies (CSIT), 2018. 270–3. doi: 10.1109/stc-csit.2018.8526717

[14] Hnatushenko V, Bulana T, Molodets B, Boldyriev D. Development of UAV Image Processing Algorithms for Early Detection of Fires in Natural Ecosystems. In: 2024 IEEE 7th International Conference on Actual Problems of Unmanned Aerial Vehicles Development (APUAVD), 2024. 219–23. doi: 10.1109/apuavd64488.2024.10765845

[15] Tucker CJ. Red and photographic infrared linear combinations for monitoring vegetation. Remote Sensing of Environment. 1979;8(2):127–50. doi: 10.1016/0034-4257(79)90013-0

[16] Wuyun D, Duan M, Sun L, Crusiol LGT, Wu N, Chen Z. Pixel-wise parameter assignment in LandTrendr algorithm: enhancing cropland abandonment monitoring using satellite-based NDVI time-series. Comput Electron Agric. 2024;227:109541. doi: 10.1016/j.compag.2024.109541

[17] Deng J, Guo Y, Chen X, Liu L, Liu W. Abandoned farmland extraction and feature analysis based on multi-sensor fused NDVI time series – A case study in western Mianchi County. Appl Sci. 2024;14:2102. doi: 10.3390/app14052102

[18] Xie Y, Spawn-Lee SA, Radeloff VC, Yin H, Robertson GP, Lark TJ. Cropland abandonment between 1986 and 2018 across the United States: spatiotemporal patterns and current land uses. Environ Res Lett. 2024;19:044009. doi: 10.1088/1748-9326/ad2d12

[19] Hong C, Prishchepov AV, Jin X, Zhou Y. Mapping cropland abandonment and distinguishing from intentional afforestation with Landsat time series. International Journal of Applied Earth Observation and Geoinformation. 2024;127:103693. doi: 10.1016/j.jag.2024.103693

[20] Wang J, Guan Y, Wang H, Zhou W. Identifying and monitoring of abandoned farmland in key agricultural production areas on the Qinghai‒Tibet Plateau: A case study of the Huangshui Basin. J Environ Manage. 2024;354:120380. doi: 10.1016/j.jenvman.2024.120380 38401505

[21] Xu S, Xiao W, Yu C, Chen H, Tan Y. Mapping Cropland Abandonment in Mountainous Areas in China Using the Google Earth Engine Platform. Remote Sensing. 2023;15(4):1145. doi: 10.3390/rs15041145

[22] Wallace CSA, Thenkabail P, Rodriguez JR, Brown MK. Fallow-land algorithm based on neighborhood and temporal anomalies (FANTA) to map planted versus fallowed croplands using MODIS data. GISci Remote Sens. 2017;54:258–82. doi: 10.1080/15481603.2017.1290913

[23] Zhang S, Zhang Y, Zhang X, Miao C, Liu S, Liu J. Revealing the distribution and change of abandoned cropland in Ukraine based on dual period change detection method. Sci Rep. 2025;15(1):5765. doi: 10.1038/s41598-025-89556-2 39962189 PMC11833136

[24] Cai X, Wang Y, Luo W, Wu Y, Cheng A, Chen J, et al. Characteristics of Tree Growth at the Early Stage of Natural Succession on Abandoned Farmland in Southwest China’s Karst Region. Forests. 2025;16(4):674. doi: 10.3390/f16040674

[25] Shiri Z, Frija A, Rejeb H, Ouerghemmi H, Le QB. Data on the Land Cover Transition, Subsequent Landscape Degradation, and Improvement in Semi-Arid Rainfed Agricultural Land in North–West Tunisia. Data. 2024;9(8):96. doi: 10.3390/data9080096

[26] Manna P, Agrillo A, Bancheri M, Di Leginio M, Ferraro G, Langella G, et al. A Geospatial Decision Support System for Supporting the Assessment of Land Degradation in Europe. Land. 2024;13(1):89. doi: 10.3390/land13010089

[27] Sakellariou M, Psiloglou BE, Giannakopoulos C, Mylona PV. Integration of Abandoned Lands in Sustainable Agriculture: The Case of Terraced Landscape Re-Cultivation in Mediterranean Island Conditions. Land. 2021;10(5):457. doi: 10.3390/land10050457

[28] Ruwanza S. The Edge Effect on Plant Diversity and Soil Properties in Abandoned Fields Targeted for Ecological Restoration. Sustainability. 2018;11(1):140. doi: 10.3390/su11010140

[29] Hardt E, Pereira-Silva EFL, Dos Santos RF, Tamashiro JY, Ragazzi S, Lins DB da S. The influence of natural and anthropogenic landscapes on edge effects. Landscape and Urban Planning. 2013;120:59–69. doi: 10.1016/j.landurbplan.2013.08.014

[30] Danso Marfo T, Datta R, Vranová V, Ekielski A. Ecotone Dynamics and Stability from Soil Perspective: Forest-Agriculture Land Transition. Agriculture. 2019;9(10):228. doi: 10.3390/agriculture9100228

[31] Banks‐Leite C, Ewers RM. Ecosystem Boundaries. Encyclopedia of Life Sciences. Wiley. 2009. doi: 10.1002/9780470015902.a0021232

[32] Suding KN, Gross KL, Houseman GR. Alternative states and positive feedbacks in restoration ecology. Trends Ecol Evol. 2004;19(1):46–53. doi: 10.1016/j.tree.2003.10.005 16701225

[33] Dutoit T, Buisson E, Gerbaud E, Roche P, Tatoni T. The status of transitions between cultivated fields and their boundaries: ecotones, ecoclines or edge effects?. Acta Oecologica. 2007;31(2):127–36. doi: 10.1016/j.actao.2006.03.010

[34] Liu Y, Zhang L, Wei X, Xie P. Integrating the spatial proximity effect into the assessment of changes in ecosystem services for biodiversity conservation. Ecol Indic. 2016;70:382–92. doi: 10.1016/j.ecolind.2016.06.019

[35] Gomes E, Banos A, Abrantes P, Rocha J. Assessing the Effect of Spatial Proximity on Urban Growth. Sustainability. 2018;10(5):1308. doi: 10.3390/su10051308

[36] North HC, Pairman D, Belliss SE. Boundary Delineation of Agricultural Fields in Multitemporal Satellite Imagery. IEEE J Sel Top Appl Earth Observations Remote Sensing. 2019;12(1):237–51. doi: 10.1109/jstars.2018.2884513

[37] Belgiu M, Csillik O. Sentinel-2 cropland mapping using pixel-based and object-based time-weighted dynamic time warping analysis. Remote Sensing of Environment. 2018;204:509–23. doi: 10.1016/j.rse.2017.10.005

[38] Ma L, Liu Y, Zhang X, Ye Y, Yin G, Johnson BA. Deep learning in remote sensing applications: A meta-analysis and review. ISPRS Journal of Photogrammetry and Remote Sensing. 2019;152:166–77. doi: 10.1016/j.isprsjprs.2019.04.015

[39] Pelletier C, Webb G, Petitjean F. Temporal Convolutional Neural Network for the Classification of Satellite Image Time Series. Remote Sensing. 2019;11(5):523. doi: 10.3390/rs11050523

[40] Kozlova A, Lischenko L, Andreiev A, Lubskyi M, Lysenko A. Water occurrence mapping of Kakhovka reservoir after the dam destruction. In: Lviv, Ukraine, 2024. doi: 10.3997/2214-4609.2024510066

[41] Sergieieva K, Kavats O, Vasyliev V, Kavats Y, Kovrov O. Machine learning-based monitoring of war-damaged water bodies in Ukraine using satellite images. In: Odesa, Ukraine, 2024. https://ceur-ws.org/Vol-3790/paper37.pdf

[42] Long Y, Sun J, Wellens J, Colinet G, Wu W, Meersmans J. Mapping the Spatiotemporal Dynamics of Cropland Abandonment and Recultivation across the Yangtze River Basin. Remote Sensing. 2024;16(6):1052. doi: 10.3390/rs16061052

[43] Toroi G-I. Integration of First Person View (FPV) Unmanned Aerial Systems in Romanian Ground Forces Operations. RMT. 2024;2024(4):126–49. doi: 10.55535/rmt.2024.4.08

[44] Franch B, Cintas J, Becker-Reshef I, Sanchez-Torres MJ, Roger J, Skakun S, et al. Global crop calendars of maize and wheat in the framework of the WorldCereal project. GIScience & Remote Sensing. 2022;59(1):885–913. doi: 10.1080/15481603.2022.2079273

[45] Pasquarella VJ, Brown CF, Czerwinski W, Rucklidge WJ. Comprehensive quality assessment of optical satellite imagery using weakly supervised video learning. In: 2023 IEEE/CVF Conference on Computer Vision and Pattern Recognition Workshops (CVPRW), 2023. 2125–35. doi: 10.1109/cvprw59228.2023.00206

[46] Busygin B, Nikulin S, Sergieieva K. Solving the tasks of subsurface resources management in GIS RAPID environment. Min Miner Depos. 2019;13:49–57. doi: 10.33271/mining13.03.049

[47] Wang X, Shu L, Han R, Yang F, Gordon T, Wang X, et al. A Survey of Farmland Boundary Extraction Technology Based on Remote Sensing Images. Electronics. 2023;12(5):1156. doi: 10.3390/electronics12051156

[48] Canny J. A computational approach to edge detection. IEEE Trans Pattern Anal Mach Intell. 1986;8(6):679–98. doi: 10.1109/tpami.1986.4767851 21869365

[49] Hong R, Park J, Jang S, Shin H, Kim H, Song I. Development of a Parcel-Level Land Boundary Extraction Algorithm for Aerial Imagery of Regularly Arranged Agricultural Areas. Remote Sensing. 2021;13(6):1167. doi: 10.3390/rs13061167

[50] Nikulin S, Sergieieva K. Assessing degradation of war-affected anthropogenic landscape using Sentinel-2 images. International Journal of Remote Sensing. 2025;46(15):5732–72. doi: 10.1080/01431161.2025.2526001

[51] Nikulin SL, Sergieieva KL, Korobko OV. Assessment of war-damaged agricultural landscape degradation in Ukraine using satellite-based contrast edge changes. In: Kyiv, Ukraine, 2025. doi: 10.33271/mining13.03.049

[52] Morell-Monzó S, Estornell J, Sebastiá-Frasquet M-T. Comparison of Sentinel-2 and High-Resolution Imagery for Mapping Land Abandonment in Fragmented Areas. Remote Sensing. 2020;12(12):2062. doi: 10.3390/rs12122062

[53] Li J, Shao K, Du J, Song K, Yu W, Liang Z, et al. Comparative Analysis of Tillage Indices and Machine Learning Algorithms for Maize Residue Cover Prediction. Remote Sensing. 2024;17(1):105. doi: 10.3390/rs17010105

[54] Zhao H, Chang C, Wang Z, Zhao G. A Large-Scale Agricultural Land Classification Method Based on Synergistic Integration of Time Series Red-Edge Vegetation Index and Phenological Features. Sensors (Basel). 2025;25(2):503. doi: 10.3390/s25020503 39860872 PMC11768949

[55] Lupa-Condo NE, Lope-Ccasa FC, Salazar-Joyo AA, Gutiérrez-Rosales RO, Jellen EN, Hansen NC, et al. Phenotyping for Effects of Drought Levels in Quinoa Using Remote Sensing Tools. Agronomy. 2024;14(9):1938. doi: 10.3390/agronomy14091938

[56] Close O, Petit S, Beaumont B, Hallot E. Evaluating the Potentiality of Sentinel-2 for Change Detection Analysis Associated to LULUCF in Wallonia, Belgium. Land. 2021;10(1):55. doi: 10.3390/land10010055

[57] Bielecka E, Jenerowicz A, Pokonieczny K, Borkowska S. Land Cover Changes and Flows in the Polish Baltic Coastal Zone: A Qualitative and Quantitative Approach. Remote Sensing. 2020;12(13):2088. doi: 10.3390/rs12132088

[58] Deng F, Peng X, Cai J, Li L, Li F, Liang C, et al. Assessing the Consistency of Five Remote Sensing-Based Land Cover Products for Monitoring Cropland Changes in China. Remote Sensing. 2024;16(23):4498. doi: 10.3390/rs16234498

[59] Butt A, Shabbir R, Ahmad SS, Aziz N. Land use change mapping and analysis using Remote Sensing and GIS: A case study of Simly watershed, Islamabad, Pakistan. The Egyptian Journal of Remote Sensing and Space Science. 2015;18(2):251–9. doi: 10.1016/j.ejrs.2015.07.003

[60] Henrich V, Krauss G, Götze C, Sandow C. Entwicklung einer Datenbank für Fernerkundungsindizes. In: Bochum, Germany, 2012.

[61] Arvor D, Meirelles M, Dubreuil V, Bégué A, Shimabukuro YE. Analyzing the agricultural transition in Mato Grosso, Brazil, using satellite-derived indices. Appl Geogr. 2012;32:702–13. doi: 10.1016/j.apgeog.2011.08.007

[62] Peng D, Zhang Y, Guan H. End-to-End Change Detection for High Resolution Satellite Images Using Improved UNet++. Remote Sensing. 2019;11(11):1382. doi: 10.3390/rs11111382

[63] Peng D, Liu X, Zhang Y, Guan H, Li Y, Bruzzone L. Deep learning change detection techniques for optical remote sensing imagery: Status, perspectives and challenges. International Journal of Applied Earth Observation and Geoinformation. 2025;136:104282. doi: 10.1016/j.jag.2024.104282

[64] Zhang C, Feng Y, Hu L, Tapete D, Pan L, Liang Z, et al. A domain adaptation neural network for change detection with heterogeneous optical and SAR remote sensing images. International Journal of Applied Earth Observation and Geoinformation. 2022;109:102769. doi: 10.1016/j.jag.2022.102769

[65] Varma MP, Pavan B, Sairam Prasad KS. Change Detection in Satellite Images using Deep Learning. In: 2024 International Conference on Communication, Control, and Intelligent Systems (CCIS), 2024. 1–6. doi: 10.1109/ccis63231.2024.10931866

[66] Shi J, Yang H, Hou X, Zhang H, Tang G, Zhao H, et al. Coupling SAR and optical remote sensing data for soil moisture retrieval over dense vegetation covered areas. PLoS One. 2025;20(1):e0315971. doi: 10.1371/journal.pone.0315971 39820018 PMC11737747

[67] Li W, Liang S, Chen K, Chen Y, Ma H, Xu J, et al. AgriFM: A multi-source temporal remote sensing foundation model for Agriculture mapping. Remote Sensing of Environment. 2026;334:115234. doi: 10.1016/j.rse.2026.115234

